# Disrupted Mitochondrial Quality Control in Atherosclerotic Disease: Mitophagy and Dynamics in Focus

**DOI:** 10.1016/j.jacbts.2026.101625

**Published:** 2026-07-23

**Authors:** Taylor Wilcox, Julie Hansen, Andrew Blanker, Michael E. Widlansky

**Affiliations:** a Cardiovascular Research Center, Medical College of Wisconsin, Milwaukee, Wisconsin, USA; b Department of Pharmacology and Toxicology, Medical College of Wisconsin, Milwaukee, Wisconsin, USA; c Division of Cardiovascular Medicine, Department of Medicine, Medical College of Wisconsin, Milwaukee, Wisconsi, USA

**Keywords:** atherosclerotic disease, mitochondrial dynamics, mitophagy

## Abstract

Mitochondrial health is essential for maintaining cardiac function, and mitophagy—the selective degradation of damaged mitochondria—is central to maintenance of mitochondrial quality. In this review, we focus on the role of mitophagy in atherosclerotic disease, exploring both canonical and noncanonical pathways. We aim to highlight how proper regulation of mitophagy supports cardiac health, while imbalances in this process can contribute to the onset and progression of cardiovascular conditions. In addition, we examine the cardioprotective potential of mitophagy in the context of disease and discuss its close relationship with mitochondrial dynamics, particularly as they relate to both macrovascular and microvascular dysfunction. Finally, we identify current gaps in knowledge and outline key questions that remain for the field to address, with the goal of guiding future research in this critical area of cardiovascular biology.

## Highlights

• Cardiovascular disease is driven by unresolved mitochondrial dysfunction and energetic failure.
• This review examines mitophagy and mitochondrial dynamics in cardiac pathophysiology.
• Targeting mitochondrial dynamics and quality control pathways offer emerging therapeutic opportunities.
• Future studies must define disease-specific targets and clinically actionable biomarkers.

## Graphical Abstract

**Figure:**
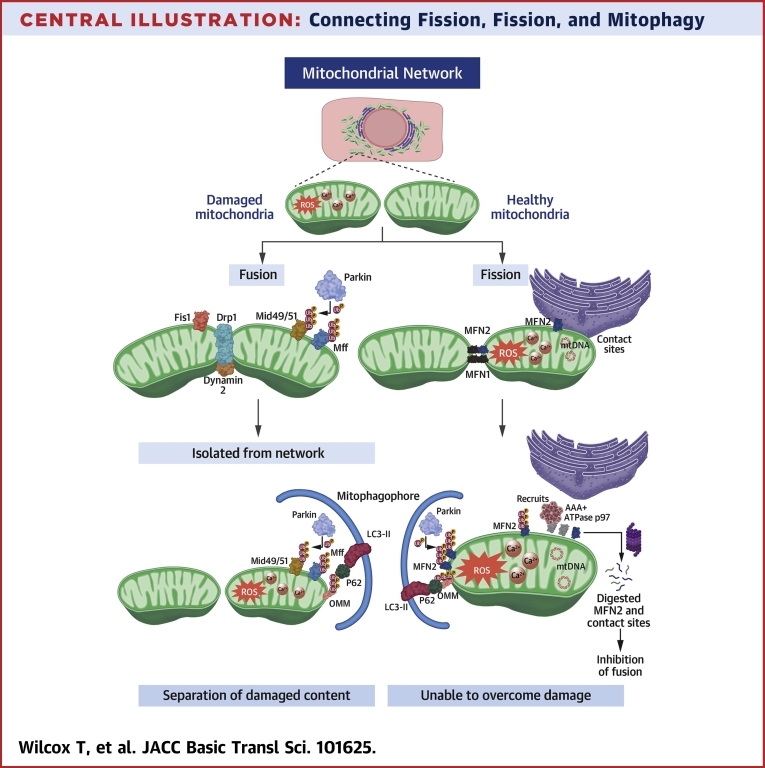

The heart is a highly metabolic organ that relies on a continuous and abundant supply of ATP to sustain its contractile function and ionic homeostasis. Similarly, vascular cells depend on efficient energy production to maintain endothelial integrity and tone, making mitochondrial function across both cardiac and vascular tissues essential for cardiovascular health. Central to this quality control is mitophagy, a specialized form of autophagy, essential for maintaining healthy, functional mitochondria. It involves the selective degradation and recycling of damaged, dysfunctional, or depolarized mitochondria to preserve mitochondrial quality and sustain optimal mitochondrial mass.^1^ Although the fundamental role of mitophagy in cellular homeostasis is well recognized, the process itself is complex—governed by distinct pathways, regulatory mechanisms, and areas of incomplete understanding that continue to evolve. Understanding how mitophagy is regulated in cardiovascular tissues, and how dysregulation contributes to pathology, is therefore essential for identifying new therapeutic targets.

In this review we provide a comprehensive synthesis of the current state of mitophagy research in atherosclerotic disease, exploring the nuances of both canonical and noncanonical pathways. We examine how the precise regulation of mitophagy supports cardiac physiology and how specific imbalances drive the onset and progression of pathology, ranging from microvascular dysfunction to heart failure. Furthermore, we integrate the interplay between mitophagy and mitochondrial dynamics, highlighting their collective impact on macrovascular and microvascular dysfunction. Unlike previous excellent reviews that examine these processes in isolation, we highlight their regulatory crosstalk and its implications for atherosclerotic disease, addressing critical gaps in current understanding.^2^, ^3^, ^4^ Finally, we identify key questions and future opportunities to translate insights from mitophagy and mitochondrial dynamics into therapeutic strategies in cardiovascular medicine.

## Mitophagy

### Canonical mitophagy

The canonical mitophagy pathway is mediated by PTEN induced kinase 1 (PINK1) and Parkin. Under normal physiological conditions, PINK1 is imported into the mitochondrial matrix via the translocase complexes, translocase of outer mitochondrial membrane (TOM) 20/22 and TOM 40 at the outer mitochondrial membrane (OMM), followed by translocase of inner mitochondrial membrane 23 at the inner mitochondrial membrane.^5^ Within the matrix, PINK1 undergoes proteolytic cleavage by mitochondrial processing peptidases, which removes its N-terminal mitochondrial targeting sequence.^6^^,^^7^ It is then further processed by the intramembrane protease presenilins-associated rhomboid-like protein (PARL), which cleaves its transmembrane domain.^8^ These sequential cleavages target PINK1 for degradation by the proteasome, preventing its accumulation under healthy conditions.^9^

In the context of mitochondrial dysfunction or disease, the loss of mitochondrial membrane potential prevents PINK1 import into the inner mitochondrial membrane, leading to its accumulation and stabilization on the OMM driven by the impaired function of the TOM complex.^10^, ^11^, ^12^ Once stabilized on the OMM, PINK1 autophosphorylates at Serine 65 and phosphorylates ubiquitin molecules on OMM proteins, creating a signal for Parkin recruitment from the cytosol.^13^, ^14^, ^15^ TBC1 domain family member 17 plays a supportive role by stabilizing PINK1 accumulation and enhancing Parkin translocation to the mitochondria.^16^^,^^17^

**Figure 1:**
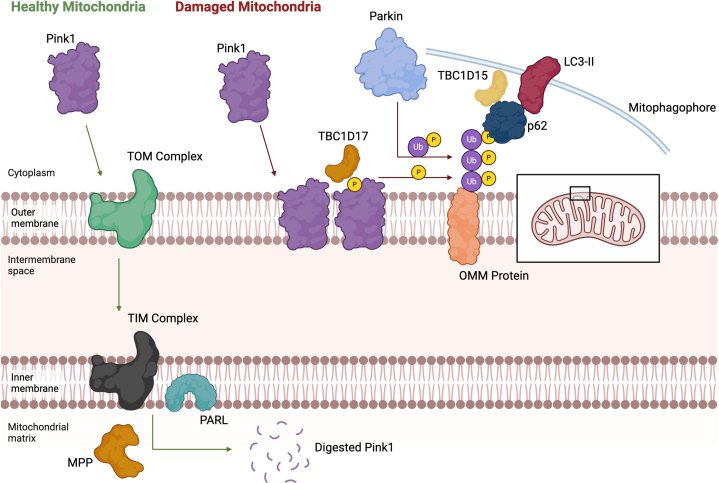
Pink/Parkin Mitophagy In healthy mitochondria, PTEN induced kinase 1 (PINK1) is imported into the organelle by first translocating across the outer mitochondrial membrane (OMM) via translocase of outer mitochondrial membrane (TOM) complexes and then through the inner mitochondrial membrane (IMM) via translocase of inner mitochondrial membrane (TIM) complexes. Once inside the matrix, PINK1 is cleaved by mitochondrial processing peptidases (MPP) and presenilins-associated rhomboid-like protein (PARL), preventing its accumulation. In damaged mitochondria, the loss of membrane potential disrupts TIM and TOM function, blocking PINK1 import and causing it to accumulate and stabilize on the OMM. Stabilized PINK1 autophosphorylates, then phosphorylates and ubiquitinates OMM proteins, signaling the recruitment of Parkin. TBC1 domain family member 17 (TBC1D17) supports both PINK1 stabilization and Parkin recruitment. Activated Parkin ubiquitinates lysine residues on PINK1-marked OMM proteins, generating polyubiquitin chains that are recognized by adaptor proteins such as p62. These adaptors bind both ubiquitin from the OMM protein and microtubule-associated protein 1 light chain 3-II (LC3-II) on the forming mitophagophore. TBC1 domain family member 15 (TBC1D15) interacts with adaptor proteins to facilitate efficient recognition and sequestration of the damaged mitochondrion into the mitophagosome.

Parkin, a cytosolic E3 ubiquitin ligase, targets several OMM proteins for ubiquitination. These include mitofusins, whose modification inhibits mitochondrial fusion; mitochondrial rho GTPase 1/2, promoting mitochondrial arrest by disrupting their attachment to microtubules; and other proteins such as voltage-dependent anion channels and components of the TOM complex.^18^^,^^19^ Notably, Parkin also ubiquitinates the same OMM proteins initially tagged by PINK1, amplifying the mitophagy signal. Parkin-mediated ubiquitination generates polyubiquitin chains on OMM proteins, which are recognized by autophagy adaptor proteins—such as sequestosome-1, also referred to as p62 (for lysine 48-linked chains), and NBR1 or optineurin (for lysine 63-linked chains).^20^ These adaptors serve dual roles, binding polyubiquitin chains and interacting with microtubule-associated protein 1 light chain 3-II (LC3-II) to facilitate mitophagosome formation. In addition, TBC1 domain family member 15 supports the recognition and sequestration of damaged mitochondria by interacting with adaptor proteins and contributing to mitophagosome recruitment (Figure 1).^17^

LC3 is initially synthesized as the precursor protein proLC3, which is cleaved by the cysteine protease autophagy-related protein 4 (ATG4) to generate LC3-I, a cytosolic form. LC3-I is then lipidated by the E2-like and E1-like enzymes ATG3 and ATG7, respectively, to form LC3-II.^21^^,^^22^ This membrane-bound form of LC3 is incorporated into the growing mitophagophore. The mitophagophore originates from a specialized region of the endoplasmic reticulum known as the omegasome, which is enriched in phosphatidylinositol 3-phosphate.^23^ Nucleation of the mitophagophore from the omegasome is initiated by 2 key signaling complexes: the Unc-51-like kinase 1 (ULK1) complex and the class III phosphoinositide 3-kinase (PI3K) complex. The ULK1 complex—comprising ULK1, ATG13, focal adhesion kinase family interacting protein of 200 kDa (FIP200), and ATG101—is tightly regulated by energy and nutrient status. Under nutrient-rich conditions, mammalian target of rapamycin complex 1 inhibits the ULK1 complex, whereas energy stress activates AMP-activated protein kinase (AMPK), which in turn activates ULK1.^24^^,^^25^ Activated ULK1 recruits the PI3K complex, composed of vacuolar protein sorting 34, Beclin-1, and ATG14, to the endoplasmic reticulum (ER) membrane.^26^, ^27^, ^28^ The PI3K complex catalyzes the production of phosphatidylinositol 3-phosphate, which acts as a docking site for proteins critical for phagophore expansion and mitophagosome maturation. Beclin-1 plays a key role in directing the PI3K complex to the ER, initiating the nucleation process.^29^

Once nucleated, the expanding phagophore is guided to the damaged mitochondria via interactions between LC3-II and adaptor proteins bound to polyubiquitin chains on the mitochondrial surface. LC3-II also drives membrane elongation and curvature, facilitating engulfment of the mitochondrion.^21^^,^^30^ Membrane expansion is further supported by vesicular contributions from the ER, Golgi apparatus, plasma membrane, and endosomes.^31^ Once the damaged mitochondrion is fully enclosed within the double-membraned structure, it is referred to as a mitophagosome.

To complete the mitophagy process, the mitophagosome must fuse with a lysosome, forming a mitolysosome. Lysosomes are typically positioned in the perinuclear region near the microtubule-organizing center.^32^^,^^33^ The mitophagosome is transported along microtubules toward the lysosome via retrograde trafficking, a process facilitated by Rab-interacting lysosomal protein. Rab-interacting lysosomal protein acts as a linker, connecting the mitophagosome to the motor protein complex composed of dynein and kinesin.^34^ On reaching the lysosome, fusion is mediated by soluble N-ethylmaleimide-sensitive factor attachment receptor (SNARE) proteins. The mitophagosome expresses syntaxin 17 and synaptosome associated protein 29, and the lysosome expresses vesicle associated membrane protein 8. These SNARE proteins interact to form a trans-SNARE complex composed of 4 α-helices that zipper together, initiating tethering, docking, and eventual membrane fusion.^35^, ^36^, ^37^ This zippering action generates tension between the mitophagosome and lysosome membranes, promoting hemi-fusion followed by pore formation. Continued assembly of the SNARE complex leads to membrane destabilization, pore expansion, and full fusion.

Once fusion is achieved, the mitophagosome releases its cargo—damaged mitochondrial contents—into the acidic environment of the lysosome. Within the mitolysosome, mitochondrial components are degraded by lysosomal hydrolases, including proteases, lipases, nucleases, and glycosidases, allowing for efficient breakdown and recycling of mitochondrial materials.

### Noncanonical mitophagy

**Figure 2:**
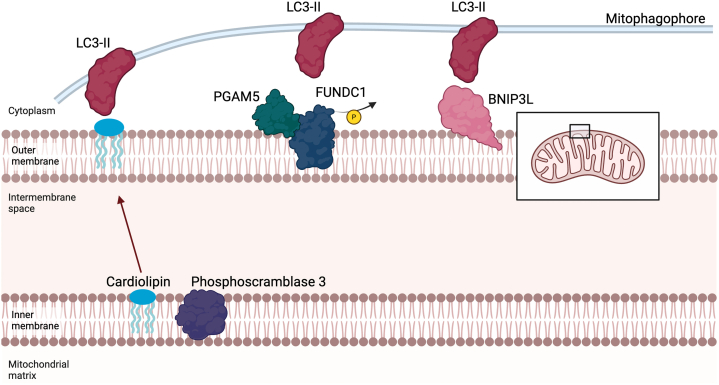
Noncanonical Mitophagy Oxidative stress activates phosphoscramblase 3, triggering cardiolipin translocation to the OMM. Cardiolipin is a direct mitophagy signal and binds to LC3-II, promoting mitophagosome formation and expansion. OMM proteins can also initiate mitophagy. FUN14 domain containing 1 (FUNDC1) can undergo dephosphorylation by phosphoglycerate mutase family member 5 (PGAM5), enhancing the binding affinity of LC3-II, promoting mitophagosome formation. Another OMM protein, NIP1-like protein X (BNIP3L), can directly bind LC3-II, promoting mitophagosome formation. Abbreviations as in Figure 1.

A notable noncanonical pathway of mitophagy involves cardiolipin-induced mitophagy. Under normal conditions, cardiolipin is predominantly localized to the inner mitochondrial membrane and its production is controlled by TAMM41 allowing for lipid composition-based regulation.^38^ However, in response to mitochondrial damage, cardiolipin is translocated to the OMM. This translocation is facilitated by oxidative stress–induced activation of phospholipid scramblase 3 and serves as an externalization signal for mitophagy initiation.^39^ Once on the outer membrane, cardiolipin functions as a direct mitophagy signal by interacting with LC3. Cardiolipin contains an LC3-interacting region motif, enabling it to bind LC3 and thereby promote mitophagosome formation and membrane expansion independently of ubiquitin signaling (Figure 2).^40^

In addition to lipid signals like cardiolipin, certain OMM proteins can directly initiate mitophagy if they possess motifs that enable interaction with components of the mitophagophore, such as LC3. One example is FUN14 domain containing 1 (FUNDC1), which, on dephosphorylation at Serine 13 by the phosphatase phosphoglycerate mutase family member 5, exhibits enhanced binding affinity for LC3, promoting mitophagosome formation.^41^ Located on the OMM, NIP1-like protein X (NIX), also known as BNIP3L, contains an LC3-interacting region motif and can induce mitophagy through direct interaction with LC3; however, NIX is highly regulated can be sequestered through interactions with anti-apoptotic Bcl-2 family proteins (Figure 2).^42^

Beyond lipid-mediated signaling pathways such as cardiolipin externalization and receptor-driven mechanisms involving proteins like FUNDC1 and NIX, additional noncanonical mitophagy pathways further expand the repertoire of mitochondrial quality control. One such pathway is mediated by Rab9-dependent, LC3-independent mitophagy. In this context, ULK1 functions as an upstream initiator of signaling, phosphorylating Rab9 at Ser179. This phosphorylation event promotes Rab9 activation and its translocation from the trans-Golgi network to damaged mitochondria. Once recruited, Rab9 facilitates the delivery of intracellular membrane sources to injured mitochondria, enabling mitophagosome formation independent of LC3.^43^ This LC3-independent mechanism provides a rapid alternative clearance route, allowing efficient removal of damaged mitochondria when canonical autophagy machinery is bypassed or limited.

Another important regulator of mitochondrial quality control is tumor necrosis factor receptor–associated factor 2 (TRAF2), which localizes to mitochondria under basal conditions to support routine mitochondrial turnover. During cellular stress, mitochondrial recruitment of TRAF2 increases significantly. Through its E3 ubiquitin ligase activity, TRAF2 tags damaged mitochondria for degradation, thereby functioning as a key quality control mediator. In addition to its role in mitochondrial clearance, TRAF2 also modulates inflammatory signaling by suppressing TLR9 expression, reducing cellular sensitivity to mitochondrial DNA released during mitochondrial damage and thereby limiting downstream inflammatory activation.^44^^,^^45^

More recently, a distinct noncanonical quality control mechanism termed heterophagy or trans-cellular mitophagy has been described.^46^ In contrast to intracellular degradation pathways, this process involves the export of damaged mitochondria from cardiomyocytes to neighboring cardiac macrophages.^47^ Cardiomyocytes package dysfunctional mitochondria into specialized vesicles known as exophers through an ATG-dependent autophagy-related process.^46^ These exophers are enriched in phosphatidylserine, which serves as an “eat-me” signal for recognition by macrophages. Neighboring macrophages detect this signal via the MER tyrosine kinase receptor, a phagocytic receptor involved in clearance of apoptotic debris.^48^ Following uptake, the mitochondrial cargo is degraded within macrophage lysosomes. This intercellular transfer of mitochondrial waste provides an alternative route of mitochondrial quality control that preserves cardiomyocyte homeostasis and helps prevent inflammasome activation in the cardiac microenvironment.^46^

## Interplay of Mitophagy With Mitochondrial Fission and Fusion Dynamics

**Figure 3:**
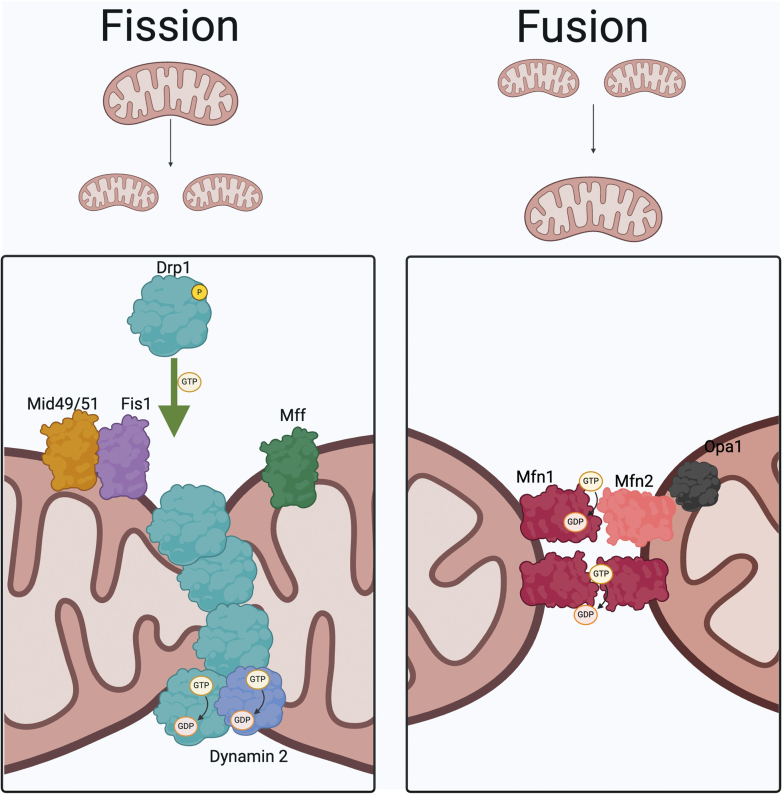
Fission and Fusion During fission, dynamin-related protein 1 (Drp1) is recruited to mitochondrial scission sites, which are marked by proteins such as mitochondrial fission factor (Mff) and mitochondrial dynamics protein 49/51 kDa (MiD49/51). Under various pathological states, mitochondrial fission protein 1 (Fis1) can also recruit Drp1. Once Drp1 is in the OMM, it undergoes conformational change via guanosine triphosphate (GTP) hydrolysis. This hydrolysis initially allows for Drp1 formation into a helical ring-like structure around the mitochondria. Further hydrolysis generates a mechanical force that constricts the mitochondria, leading to membrane scission. The process is reinforced by the recruitment of Dynamin 2, which provides additional GTPase activity to assist in completing the fission event. During fusion, microfusion proteins 1 and 2 (Mfn1 and Mfn2) form hetero- or homodimers between adjacent mitochondria. Their interactions are then driven by GTP hydrolysis, which facilitates membrane tethering and mitochondrial fusion. GDP = guanosine diphosphate; Opa1 = optic atrophy 1; other abbreviations as in Figure 1.

Proper mitochondrial dynamics, balancing fusion and fission of mitochondria are necessary for the effective segregation and elimination of damaged mitochondria.^49^, ^50^, ^51^ Balanced mitochondrial dynamics help maintain proper mitochondrial number, size, and intracellular distribution.^52^ Balanced dynamics also facilitates the removal of damaged mitochondria through mitophagy, allowing cells to rapidly adapt to metabolic and environmental changes.^53^^,^^54^ The interplay between fission and fusion proteins and mitophagy is depicted in Figure 3.

### Mitochondrial fusion

Mitochondrial fusion consists of 2 main events: 1) outer membrane fusion and 2) inner membrane fusion.^55^ Outer membrane fusion is mediated by mitofusin proteins, Mfn1 and Mfn2, which are located on the OMM.^56^^,^^57^ These proteins can form either homodimers or heterodimers between adjacent mitochondria. Their trans-interactions, driven by guanosine triphosphate (GTP) hydrolysis, facilitate membrane tethering and fusion.^58^ This process is finely regulated through mechanisms such as phosphorylation and ubiquitination and is influenced by mitochondrial calcium levels and redox states (Figure 3).^59^

Inner membrane fusion is regulated by optic atrophy 1 (Opa1), a GTPase localized to the inner mitochondrial membrane. Opa1 exists in 2 main forms: the membrane-bound long form (l-Opa1) and a soluble short form (s-Opa1), which is generated by proteolytic cleavage by the mitochondrial protease metalloendopeptidase OMA1 in the intermembrane space.^50^^,^^60^ The balance between l-Opa1 and s-Opa1 is critical for maintaining proper mitochondrial fusion and cristae structure.^50^ During fusion, l-Opa1 oligomerizes on GTP binding and mediates inner mitochondrial membrane tethering between adjacent mitochondria. Cardiolipin, a mitochondrial phospholipid, aids in the localization and stabilization of Opa1 during this process.^50^^,^^58^ The activity of Opa1 is closely tied to the mitochondrial membrane potential: depolarization triggers OMA1-mediated cleavage of l-Opa1 into s-Opa1, which inhibits fusion, and membrane hyperpolarization stabilizes l-Opa1 and promotes fusion.^59^^,^^61^

### Mitochondrial fission

Under normal physiological conditions, fission is initiated by the recruitment of dynamin-related protein 1 (Drp1) to specific scission sites on the mitochondrial surface.^62^ These sites are often marked by contact with the endoplasmic reticulum and are defined by the presence of mitochondrial fission factor, with assistance from mitochondrial dynamics protein 49 and MiD51.^56^^,^^63^ In pathological states such as hypoxia or hyperglycemia, Drp1 also can be recruited by mitochondrial fission protein 1 (Fis1).^64^

Once localized to the OMM, Drp1 binds GTP, triggering a conformational change that allows it to oligomerize into a helical ring-like structure around the mitochondrion.^65^ GTP hydrolysis then drives further conformational changes, generating mechanical force that constricts the mitochondrion, ultimately leading to membrane scission.^42^ This process is reinforced by the recruitment of Dynamin-2, which provides additional GTPase activity to assist in completing the fission event (Figure 3).^66^

### Regulatory connections of fusion and fission to mitophagy

In murine embryonic fibroblasts derived from Mfn1/Mfn2/Drp1 triple-floxed mice, adeno-Cre-mediated deletion of these fusion and fission genes led to a fragmented mitochondrial network and impaired membrane potential.^67^ When mitophagy was concurrently disrupted through loss of Parkin, damaged mitochondria were not efficiently cleared, resulting in mitochondrial accumulation and subsequent myocardial dysfunction in conditional triple knockout mice. These findings support a model in which mitochondrial dynamics serve as a prerequisite for mitophagy-mediated quality control.

Dysregulated mitochondrial dynamics involving MFN2, Drp1, and Fis1 contribute to a variety of diseases, including neurodegeneration, cardiomyopathies, and cancer.^49^^,^^50^^,^^57^ The dynamic crosstalk between MFN2-mediated mitochondrial fusion, Fis1/Drp1-driven fission, and PINK1-Parkin-dependent mitophagy forms an integrated network critical for mitochondrial quality control.^52^^,^^57^^,^^68^ Fragmentation is a prerequisite for efficient mitophagy because large, interconnected mitochondrial networks are less accessible to autophagosomes (Central Illustration).^52^ Mitochondrial fission, particularly via Drp1 and Fis1, is essential for fragmenting damaged mitochondria and promoting mitophagy, but its excessive fission activation can inhibit mitophagic clearance.^56^^,^^57^ Drp1 activity is tightly regulated through various posttranslational modifications (phosphorylation and SUMOlyation) and signaling pathways.^50^^,^^57^^,^^58^^,^^60^^,^^69^ Drp1-mediated fission ensures that damaged mitochondrial fragments are properly isolated and primed for ubiquitination and subsequent removal.^56^^,^^57^ Fis1 assists in recruiting Drp1 to fission sites and modulates mitochondrial division, thereby indirectly facilitating mitophagy initiation.^60^ Excessive or dysregulated fission paradoxically inhibits mitophagy.^52^^,^^57^ Fis1 overexpression may impair mitochondrial motility and hinder autophagic flux, thereby indirectly limiting mitophagy.^57^ The PINK1–Parkin pathway can, in turn, modulate fission machinery.^52^^,^^57^ Parkin can ubiquitinate Fis1 and Drp1, affecting their stability and activity, further linking mitochondrial dynamics to quality control.^53^

**Central Illustration:**
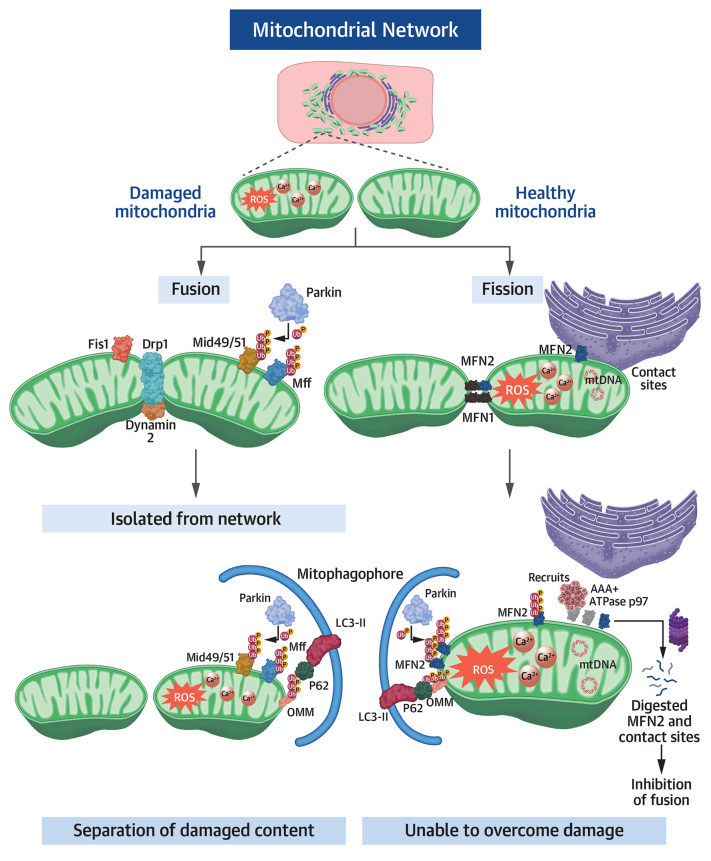
Connecting Fission, Fission, and Mitophagy

MFN2 not only mediates fusion but also acts as a mitophagy receptor regulated by posttranslational modifications.^58^^,^^69^ Fusion proteins are often suppressed during mitophagy. MFN2-driven fusion counteracts excessive fragmentation by promoting mitochondrial content mixing, which helps dilute damage and postpone mitophagy initiation.^51^ MFN2 is a preferential substrate of Parkin and undergoes rapid ubiquitination that marks MFN2 for proteasomal degradation, facilitating mitophagy progression.^54^^,^^68^ Ubiquitination of MFN2 not only marks it for proteasomal degradation but also orchestrates remodeling of ER-mitochondria contacts by recruiting the AAA + ATPase p97, which disassembles tethering complexes. This remodeling is a required for isolating damaged mitochondria before autophagosome engulfment.^68^^,^^69^

The balanced interplay of fission, fusion, and mitophagy ensures mitochondrial integrity, metabolic flexibility, and cellular health, with disruption contributing to diverse pathologies.^50^^,^^57^^,^^66^ We will review the state of our current knowledge of the role of mitophagy in the pathogenesis of cardiovascular diseases in the next section, keeping in mind the critical interplay of mitophagy with fusion and fission regulation.

## Mitophagy in Atherosclerotic and Coronary Microvascular Disease

Mitochondrial health and maintenance play a critical role in the development, progression, and treatment of various atherosclerotic diseases, largely through the regulation of mitophagy. As a key quality control mechanism, mitophagy ensures the removal of damaged mitochondria to preserve cellular function. However, its activity must be tightly regulated—both insufficient and excessive mitophagy can disrupt cellular homeostasis, highlighting the need for a balanced response (Table 1).

**Table 1:** Mitophagy Pathway Changes and Consequences in Coronary and Peripheral Arterial Disease and Coronary Microvascular Disease

Cardiovascular Disease	Model	Intervention	Observed Effect	Mitophagy Pathway Involved	Functional Outcome	Reference
Coronary artery disease	HAEC	Ox-LDL exposure leading to increased NR4A1	Excessive mitophagy leading to loss of mitochondrial mass	PINK1/Parkin	Overactivation of mitophagy impaired energy metabolism	86
Coronary artery disease	HUVEC	Salvianolic acid B	Downregulated ROCK1-mediated mitophagy under high glucose + ox-LDL	ROCK1-linked	Reduced mitochondrial ROS and EC apoptosis	87
Coronary microvascular disease	Diabetic mice	L-carnitine	Restored PINK1/Parkin mitophagy vis PARL inhibition	PINK1/Parkin	Improved microvascular integrity, reduced inflammation	72
Coronary microvascular disease	Mice	Empagliflozin	Activated AMPKα/ULK1/FUNDC1 mitophagy	FUNDC1	Protected endothelium from I/R injury	73
Peripheral artery disease	Human	Observation	PAD patients showed accumulation of ETC, decreased p62, increased LC3, impairing mitophagy	PINK1/Parkin	Impaired mitophagy, reduced oxidative capacity, and worse physical performance in PAD	88
Peripheral artery disease	Human	Observation	Muscle fibers displayed aberrant mitophagy	Undifferentiated	Aberrant mitophagy in calf muscle fibers linked to impaired walking endurance in PAD	89
Peripheral artery disease	Mice	Autophagy inhibition	Blocked mitophagy caused mitochondrial accumulation leading to atrophy	ATG-dependent	Muscle wasting and dysfunction	90

ATG = autophagy-related protein; AMPK = AMP-activated protein kinase; EC = endothelial cell; ETC = electron transport chain; FUNDC1 = FUN14 domain containing 1; HAEC = human aortic endothelial cells; HUVEC = human umbilical vein endothelial cell; I/R = ischemia/reperfusion; LC3 = microtubule-associated protein 1 light chain 3; NR4A1 = nuclear receptor subfamily 4 group A member 1; ox-LDL = oxidized low-density lipoprotein; PAD = peripheral artery disease; PARL = presenilins-associated rhomboid-like protein; PINK1 = PTEN induced kinase 1; ROCK1 = rho-associated protein kinase 1; ROS = reactive oxygen species; ULK1 = Unc-51-Like Kinase 1.

### Coronary microvascular disease

Coronary microvascular disease (CMVD) is characterized by impaired vasodilation and vasoconstriction of coronary resistance vessels, which can lead to a clinical syndrome that includes chest pain due to inadequate myocardial perfusion.^70^^,^^71^ Emerging evidence suggests that mitophagy plays a crucial role in protecting the microvascular endothelium under pathological conditions such as CMVD. For instance, in a mouse model of type 2 diabetes mellitus with coronary microvascular dysfunction, L-carnitine treatment improved coronary microvascular endothelial health and reduced vascular inflammation. In cultured mouse coronary microvascular endothelial cells, diabetic conditions impaired PINK1/Parkin-mediated mitophagy, which was restored by L-carnitine through inhibition of PARL detachment. However, when PARL was overexpressed, mitophagy was suppressed, and L-carnitine no longer conferred protective effects on mitochondrial integrity or coronary microvascular function.^72^ In another study, the antidiabetic drug empagliflozin activated the FUNDC1-dependent mitophagy pathway in coronary microvascular endothelial cells during ischemia/reperfusion injury in mice. Importantly, endothelial-specific deletion of FUNDC1 abolished the beneficial effects of empagliflozin, highlighting that mitophagy is essential for its endothelial protection.^73^ These early findings support the hypothesis that mitophagy may be downregulated during CMVD and that its activation could offer microvascular protective effects across multiple pathological contexts, particularly ischemia with no obstructive coronary artery disease (CAD).

### Coronary artery disease

CAD is primarily driven by the progression of atherosclerosis, a chronic inflammatory condition affecting the arterial walls. In the setting of CAD, impaired mitophagy contributes to mitochondrial dysfunction, resulting in elevated reactive oxygen species (ROS), increased vascular inflammation, and cardiomyocyte death.^74^, ^75^, ^76^, ^77^ Thus, although atherosclerosis drives the structural basis of CAD, disrupted mitophagy may exacerbate disease progression by amplifying oxidative stress and inflammatory damage.

**Figure 4:**
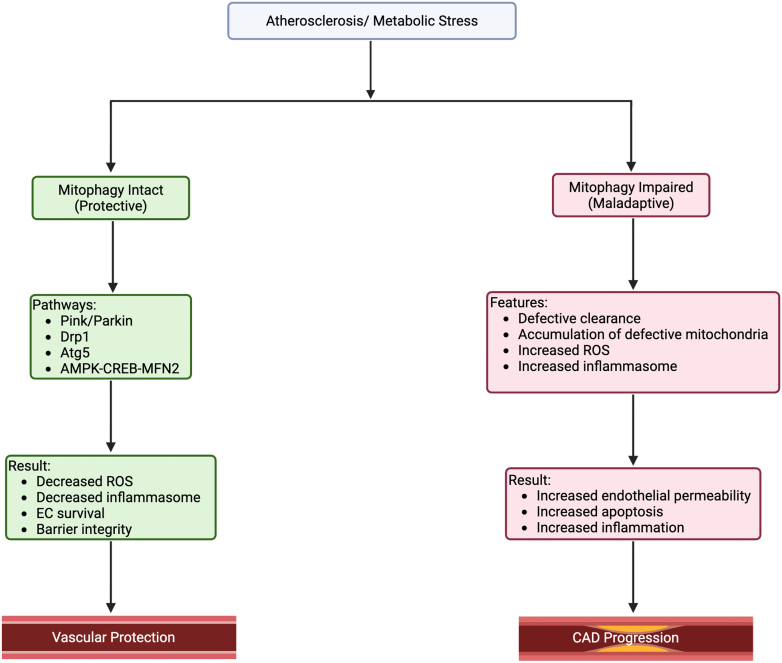
Coronary Artery Disease and Mitophagy Mitophagy supports vascular health by clearing damaged mitochondria, thereby promoting endothelial cell (EC) survival and maintaining barrier integrity in the setting of metabolic stress or early atherosclerosis. Effective mitophagy preserves mitochondrial function and limits inflammatory signaling, contributing to protection against coronary artery disease (CAD) progression. Conversely, impaired mitophagy leads to the accumulation of dysfunctional mitochondria, triggering inflammation and excessive reactive oxygen species (ROS) production. These events increase endothelial permeability, promote cell death, and ultimately accelerate CAD progression. AMPK = AMP-activated protein kinase; ATG5 = autophagy-related 5; CREB = cAMP response element-binding protein; DRP1 = dynamin-related protein 1; other abbreviation as in Figure 3.

Mitophagy may play a role in suppressing the early development of CAD by reducing endothelial cell (EC) permeability. In rat coronary microvascular cells, activation of dynamin-related protein 1 (DRP1) increased PINK1/Parkin-mediated mitophagy and protected against lipopolysaccharide-induced EC hyperpermeability.^78^ Similarly, autophagy-related 5 (ATG5)-null mice demonstrated increased atherosclerotic plaque burden and elevated inflammasome markers, suggesting that basal mitophagy is atheroprotective and that impaired mitophagy may promote atherosclerosis through inflammasome hyperactivation.^75^ In vitro studies further support the atheroprotective role of mitophagy. Human umbilical vein endothelial cells (HUVECs) exposed to palmitic acid showed a significant increase in PINK1/Parkin-mediated mitophagy, which prevented palmitic acid–induced mitochondrial dysfunction, ROS production, and apoptosis. These findings align with a study that found the arteries of obese mice had increased PINK1/Parkin expression in vascular endothelial cells under metabolic stress, implicating that this pathway may be protective.^79^ In addition, knockdown of CR6-interacting factor 1 (CRIF1) in HUVECs led to mitochondrial dysfunction and increased expression of the redox protein p66Shc, a mediator of mitochondrial oxidative stress and metabolic memory. This, in turn, activated mitophagy, which reduced ROS levels and subsequently downregulated p66Shc, revealing a negative feedback loop in which mitophagy limits oxidative stress.^80^, ^81^, ^82^ Further studies in HUVECs suggest that PTEN inhibition promotes EC survival during oxidized low-density lipoprotein (ox-LDL)-induced injury by activating the AMPK–cAMP response element-binding protein–Mitofusion 2–mitophagy signaling, underscoring another route by which mitophagy supports endothelial health.^83^ Collectively, these studies suggest a critical role of mitophagy in defending against early atherosclerosis stressors (Figure 4).

Mitophagy also plays a role in later stages of CAD, particularly under ischemic stress and in advanced atherosclerotic lesions. Inhibition of FUNDC1-mediated mitophagy leads to increased mitochondrial apoptosis in both coronary microvascular endothelial cells and cardiomyocytes, whereas restoration of FUNDC1 activity confers a survival advantage during ischemia-reperfusion injury.^74^^,^^77^^,^^84^ Moreover, increased autophagic flux has been shown to attenuate plaque progression in advanced atherosclerosis, whereas ATG5-and nicotinamide adenine dinucleotide phosphate oxidase 2–deficient macrophages exhibited increased apoptosis and oxidative stress due to elevated nicotinamide adenine dinucleotide phosphate oxidase activity, contributing to necrotic core formation and plaque instability.^85^

Although mitophagy is generally protective in CAD, excessive or dysregulated mitophagy may contribute to disease progression. For example, elevated levels of nuclear receptor subfamily 4 group A member 1, induced by ox-LDL, have been shown to stimulate PINK1/Parkin-mediated mitophagy in human aortic endothelial cells. This led to a pathological reduction in mitochondrial mass, resulting in impaired energy production and mitochondrial dysfunction.^86^ Similarly, in HUVECs, salvianolic acid B was found to inhibit ox-LDL and high glucose-induced activation of rho-associated protein kinase 1–mediated mitophagy, which in turn reduced mitochondrial ROS in endothelial cells associated with vascular failure.^87^ However, neither study conclusively demonstrated that increased mitophagy drives endothelial dysfunction, and these observed changes may instead reflect insufficient compensatory responses. Future studies should directly manipulate mitophagy to determine whether it plays a causal role.

### Peripheral artery disease

Peripheral artery disease (PAD) is characterized by impaired blood flow to the limbs. Although direct data regarding peripheral vasculature is limited, extrapolating from the established role of mitophagy in coronary atherosclerosis suggests that dysregulated mitophagy likely contributes to the macrovascular atherosclerosis driving PAD. Downstream of vessel pathology, mounting evidence suggests that dysregulated mitophagy contributes to mitochondrial dysfunction in affected skeletal muscle fibers. In gastrocnemius myofibrils from patients with PAD, lower ankle-brachial index values were associated with the accumulation of electron transport chain complexes, an increased ratio of lipidated LC3, and decreased p62 levels, consistent with impaired mitophagy accompanied by compensatory activation of macroautophagy.^88^ This disruption in mitochondrial quality control correlates with diminished oxidative capacity and functional decline in skeletal myocytes. Muscle fibers from PAD patients demonstrate an accumulation of LC3 within the sarcolemma and perinuclear regions of myofibers, indicating stalled or incomplete mitophagy, which may underlie reduced walking performance and progressive muscle dysfunction.^89^ Supporting this, inhibition of mitophagy in rodent skeletal muscle cells leads to the accumulation of dysfunctional mitochondria and subsequent muscle atrophy.^90^ At the molecular level, transcriptomic and proteomic analyses of gastrocnemius muscles from patients with PAD reveal aberrant stoichiometric ratios of mitochondrial respiratory proteins compared with non-PAD controls. In non-PAD muscle, greater mitochondrial respiratory protein abundance correlated with higher complex II and IV activity within oxidative myofibers, whereas this relationship was lost in PAD muscle, which also exhibited reduced levels of rate-limiting enzymes such as hexokinase and pyruvate kinase. These data suggest that defective mitophagy prevents the clearance of damaged mitochondrial components, thereby contributing to abnormal respiratory activity and triggering hypoxia-adaptive responses within ischemic muscle tissue.^91^

## Current State of Our Knowledge and Future Opportunities

Mitophagy is a key quality control mechanism that removes damaged mitochondria to maintain cellular homeostasis in the heart and vasculature. Under physiological conditions, it preserves ATP production, limits oxidative stress, and supports cardiomyocyte survival. Impaired mitophagy is increasingly recognized as a central contributor to mitochondrial dysfunction atherosclerotic disease.^92^ Mitochondrial fission facilitates segregation of dysfunctional mitochondria, allowing targeted removal through mitophagy, whereas fusion helps maintain mitochondrial integrity and bioenergetic efficiency. Disruptions in either process alter the pool of mitochondria available for degradation, linking aberrant fission–fusion states to insufficient or maladaptive mitophagy (Central Illustration). This dynamic interplay underscores that mitophagy often acts downstream of altered mitochondrial morphology.^93^ Defective or dysregulated mitophagy contributes to hallmark atherosclerotic pathologies, including energy imbalance, elevated ROS, mitochondrial DNA damage, and impaired calcium handling. In stress conditions such as myocardial infarction, heart failure, and ischemia-reperfusion injury, inadequate mitophagic clearance amplifies cardiomyocyte injury and worsens remodeling and functional decline.^94^^,^^95^ These findings highlight mitophagy as both a biomarker of mitochondrial health and a promising therapeutic target for cardiovascular intervention.

Although there has been considerable growth in our knowledge of the regulation of mitophagy, our understanding remains incomplete. Although the PINK1/Parkin pathway remains the most extensively characterized mechanism of mitophagy, alternative pathways are less well understood. Key questions remain, including what factors determine the selection of one mitophagy pathway over another, whether these pathways operate cooperatively or independently, and whether specific pathways are preferentially engaged in certain cell types or pathological contexts. These knowledge gaps are detailed in Table 2. Elucidating the upstream signals and molecular regulators that govern pathway selection may reveal mechanisms that can be selectively targeted to enhance mitophagy in disease states while minimizing unintended effects on other cellular processes. Further research is also needed to elucidate how these diverse mitophagy mechanisms are regulated and integrated under physiological and disease conditions.

**Table 2:** Key Unanswered Questions

Key Question	Rationale
What factors determine the selection of one mitophagy pathway over another?	• Clarification of the stress signals that dictate pathway engagement • Identification of molecular regulators that determine canonical vs noncanonical activation • Potential to establish biomarkers reflective of pathway utilization and disease state
Are these pathways operating cooperatively or independently?	• Enables deeper mechanistic understanding of mitophagy regulation • Creates opportunities for the development of pathway-selective therapeutic strategies
Are specific pathways are preferentially engaged in certain cell types or pathological contexts?	• Provides insight into disease-specific mechanisms of mitochondrial quality control • Opens opportunities for precision medicine approaches that target the most relevant pathways

In addition, the dynamic balance among mitochondrial fission, fusion, and mitophagy—particularly under stress conditions such as ischemia or metabolic overload—remains incompletely understood. Identifying the dominant regulators that orchestrate this balance during cardiovascular stress could yield promising therapeutic targets aimed at restoring mitochondrial quality control and improving cardiac function. Such targets may offer a more refined approach to treatment by selectively modulating mitochondrial turnover while preserving essential mitochondrial functions.

Additional work is also necessary to determine how mitochondrial biogenesis is coordinated with mitophagy and dynamics to maintain mitochondria health. The extent to which mitochondrial fission, fusion, and mitophagy are coupled with mitochondrial biogenesis remains unclear. Understanding this relationship is essential for developing strategies that not only clear dysfunctional mitochondria but also promote the renewal of a healthy mitochondrial network, particularly in the setting of chronic cardiac stress (Table 3).

**Table 3:** Future Directions

Knowledge Gap	Key Question	Rationale	Potential Approach
The factors that determine whether canonical or noncanonical mitophagy predominates under different physiological and pathological conditions remain unclear.	What upstream signals and molecular regulators govern mitophagy pathway selection across diverse cellular contexts?	Defining the mechanisms of pathway selection may reveal opportunities to selectively enhance mitophagy in cardiovascular disease, promoting mitochondrial quality control in stressed cardiac and vascular tissues while minimizing unintended effects on other cellular processes.	• Conditional knockouts • CRISPR-based screening • Validation of potential regulators in disease-relevant in vivo models
The interplay between mitochondrial fission, fusion, and mitophagy—especially during stress conditions such as ischemia or metabolic overload—is not fully understood.	Which regulators coordinate the balance between mitochondrial dynamics and mitophagy during cardiovascular stress?	Clarifying how these processes are integrated may identify therapeutic targets that restore mitochondrial quality control in cardiovascular disease while preserving essential mitochondrial function.	• Employ live-cell imaging, genetic manipulation of mitochondrial dynamics proteins (eg, DRP1, OPA1, MFN1/2), and in vivo models of cardiovascular stress to dissect the regulators that orchestrate mitochondrial turnover
How mitochondrial biogenesis is coordinated with mitophagy and mitochondrial dynamics to maintain quality control remains poorly understood, particularly in the context of chronic cardiac stress.	To what extent is mitochondrial biogenesis coupled with mitophagy, and how does this relationship contribute to the renewal of a functional mitochondrial network?	Defining this coupling is critical for developing strategies that both eliminate dysfunctional mitochondria and promote the regeneration of healthy organelles, thereby preserving cardiac function under chronic stress conditions.	• Use genetic and pharmacological tools to modulate key regulators of biogenesis (eg, PGC-1α, NRF1/2, TFAM) alongside mitophagy pathways, combined with metabolic and functional assessments in models of chronic cardiac stress

DRP1 = dynamin-related protein 1; MFN1/2 = microfusion proteins 1 and 2; NRF1/2 = Nuclear Respiratory Factors 1 and 2; OPA1 = optic atrophy 1; PGC-1α = peroxisome proliferator-activated receptor-gamma coactivator 1-alpha; TFAM = Mitochondrial Transcription Factor A.

Although we have a growing body of in vitro and preclinical animal model data regarding the role of mitophagy in a wide variety of cardiovascular diseases, mechanistic data from studies in human tissues and in vivo work linking human disease or disease surrogates to mitophagy remain limited. Several groups have studied urolithin-A, a metabolite arising from the gut microbiota–mediated transformation of ellagitannins and a mitophagy activator, in older adults to determine its effects on cardiovascular performance, with mixed results.^96^^,^^97^ Urolithin-A's relationship to the gut microbiota reinforces the growing concept that the activity of the gut microbiota influences cardiovascular health.^98^^,^^99^ Both urolithin-A and mitoquinone mesylate, a mitochondrial-targeted antioxidant, have shown favorable effects on vascular function in older adults.^100^, ^101^, ^102^ However, neither study assessed the effects of their interventions on mitophagy. To our knowledge, there are no human data on the effects of in vivo mitophagy activation on cardiovascular health in individuals with the disease states described previously in this review. A key limitation for the study of the effects of mitophagic modulation in humans is the lack of approved, targeted mitophagy modulators. MTK458 and FB231, PINK1 activators, are under development and currently in preclinical studies focusing primarily on neurodegenerative diseases. MTX652, a ubiquitin-specific protease 30 inhibitor that stimulates PINK/Parkin-mediated mitophagy by inhibiting deubiquitination, is in phase 2 studies evaluating acute kidney injury, with plans to assess its impact on heart failure with preserved ejection fraction.

Given the connections between mitophagy and mitochondrial fusion and fission, small molecules and pharmacological agents under development to modulate this axis could also regulate mitophagy. However, to date, most of these molecules have served mainly as preclinical tool compounds rather than developed as therapeutics. Mitochondrial division inhibitor-1 has commonly been described as a Drp1 inhibitor, although recent data suggest its mechanism of action is more likely mitochondrial complex I inhibition.^103^ A covalent Drp1-inhibitor, mitochondrial division inhibitor has recently been reported, but little is known about its biological effects. Echinacoside, which promotes mitochondrial fission by targeting casein kinase 2, appears to have favorable effects in stroke in preclinical models.^104^^,^^105^ Small-molecule activators of Mfn2 have also been tested in preclinical neurodegenerative disease models.^106^

The current developmental landscape and the clear intersections of dysfunctional mitophagy and related mitochondrial dynamics with atherosclerotic diseases in in vitro and preclinical models suggest we have an opportune moment to test emerging mitophagy-focused interventions in human cardiovascular tissues and models. Many of the agents currently in development could be repurposed to focus on their cardiovascular effects. Testing in intact human vessel models using pharmacological and molecular approaches, along with human cardiac and vascular organoid and organ-on-a-chip models, could accelerate our understanding of what interventions may have cardiovascular benefit, as well as de-risk the development of drugs toward these targets by increasing the likelihood that they will have similar biological activity when they finally reach phase 1 and 2 studies.^107^

The opportunity to bring mitochondrial medicines for atherosclerotic disease appears on the horizon. Targeting mitophagy appears to be a promising avenue for therapeutic development, providing significant opportunities for researchers in this field.

## Funding Support and Author Disclosures

Dr Widlansky is supported by HL144098, HL173778, K24HL152143, R38HL167238, KL2TR001438, and AHA9639591. Ms Wilcox is supported by 25PRE1360839. Dr Widlansky has an ownership stake in Sanacor, a company focused on developing mitochondrial fission and fusion inhibitors to treat atherosclerotic and microvascular diseases. All other authors have reported that they have no relationships relevant to the contents of this paper to disclose.
